# Supplementary material for: Single-cell multi-omic analyses resolve the cellular diversity of ALK/ROS1/MET/NTRK-fused gliomas in infants and older children
Source: bioRxiv. 2026 Jul 16:2026.07.16.738862. Preprint. [Version 1] doi: 10.64898/2026.07.16.738862 (PMC13404746; doi:10.64898/2026.07.16.738862)
Supplement: Supplement 1 [file NIHPP2026.07.16.738862v1-supplement-1.pdf]

bioRxiv preprint doi: https://doi.org/10.64898/2026.07.16.738862; this version posted July 16, 2026. The copyright holder for this preprint (which was not certified by peer review) is the author/funder, who has granted bioRxiv a license to display the preprint in perpetuity. It is made available under a CC-BY 4.0 International license.

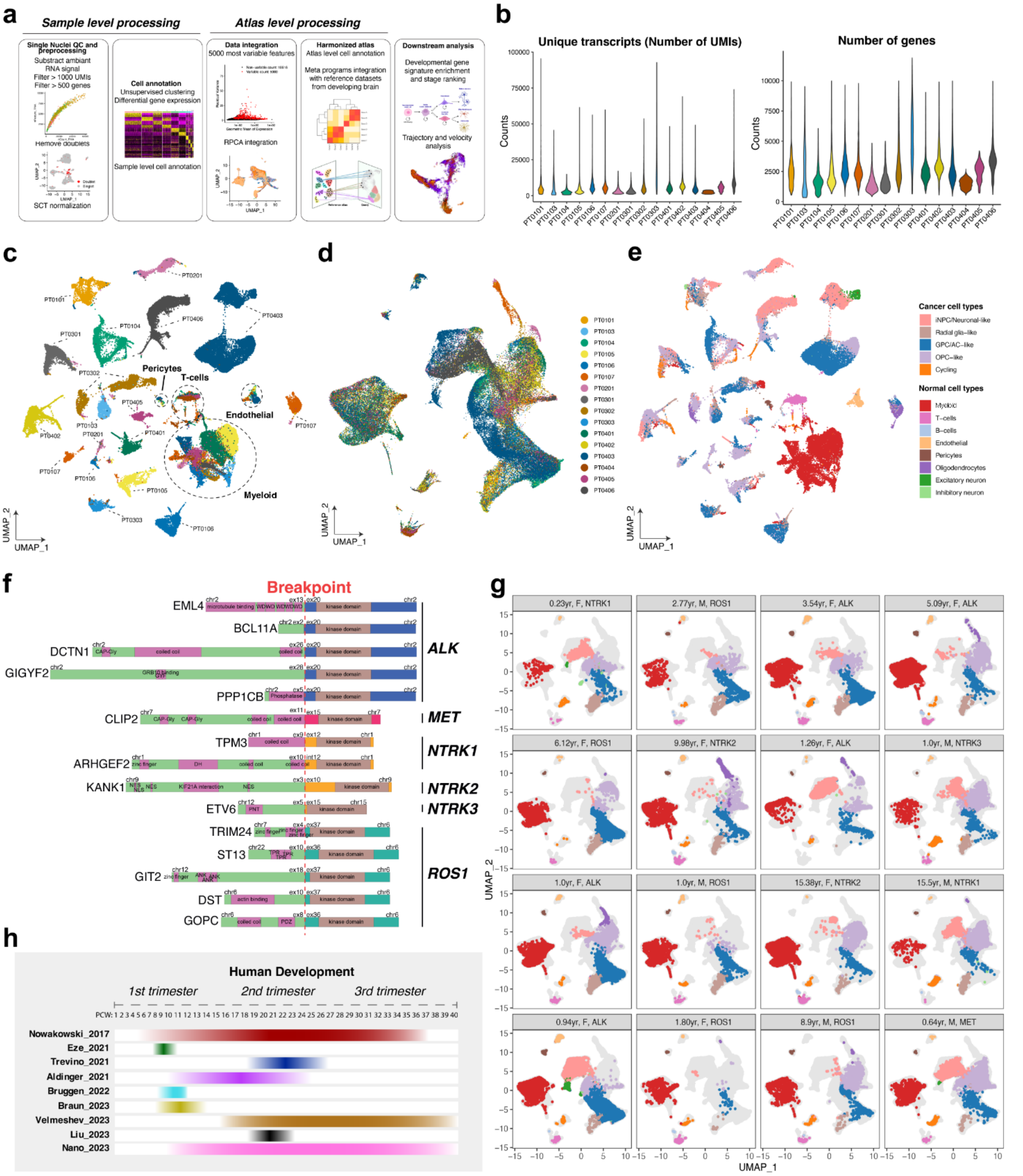

Figure S1– Single-nucleus RNA-seq analysis pipeline. (a) Data processing workflow. (b) Distribution of the number of unique transcripts (UMIs) and number of genes identified in individual nuclei per patient sample. (c) UMAP of unintegrated data (no batch correction) colored by patient. (d) UMAP of batch corrected data colored by patient. (e) UMAP of unintegrated data colored by cell type. (f) Main patient RTK gene fusion and breakpoints. (g) Integrated UMAP embeddings of single-nucleus transcriptome from individual patients (10X Multiome, n=16) and color-coded by cell type. (h) Developmental datasets used for data integration (see also Fig. S3) with the corresponding developmental windows

bioRxiv preprint doi: https://doi.org/10.64898/2026.07.16.738862; this version posted July 16, 2026. The copyright holder for this preprint (which was not certified by peer review) is the author/funder, who has granted bioRxiv a license to display the preprint in perpetuity. It is made available under a CC-BY 4.0 International license.

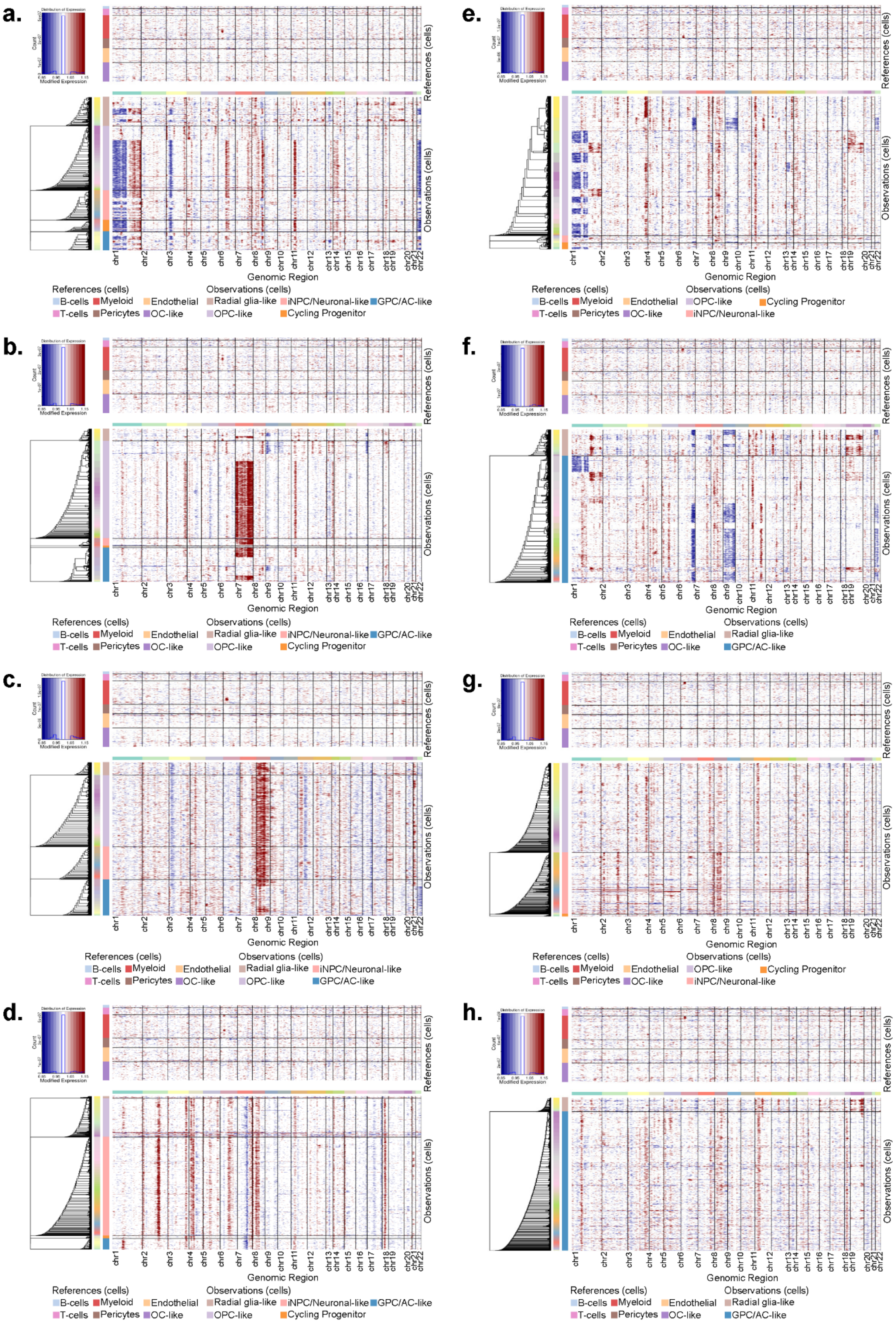

bioRxiv preprint doi: https://doi.org/10.64898/2026.07.16.738862; this version posted July 16, 2026. The copyright holder for this preprint (which was not certified by peer review) is the author/funder, who has granted bioRxiv a license to display the preprint in perpetuity. It is made available under a CC-BY 4.0 International license.

Figure S2- Chromosomal copy number variation inferred from single-cell RNAseq. For all analyses, inferCNV reference profiles were constructed using T cells, B cells, myeloid, endothelial cells, pericytes, and oligodendrocyte-like (OC-like) cells. Heatmaps indicate inferred copy number gains and losses across individual cells. (a) NTRK1 samples. (b) NTRK2 samples. (c) NTRK3 samples. (d) MET samples. (e) ROS1 samples restricted to OPC-like, iNPC/Neuronal-like cells and Cycling Progenitor cells. (f) ROS1 samples restricted to radial glia-like and GPC/astrocyte-like cells. (g) ALK samples restricted to OPC-like, iNPC/Neuronal-like cells and Cycling Progenitor cells. (h) ALK samples restricted to radial glia-like and GPC/astrocyte-like cells.

bioRxiv preprint doi: https://doi.org/10.64898/2026.07.16.738862; this version posted July 16, 2026. The copyright holder for this preprint (which was not certified by peer review) is the author/funder, who has granted bioRxiv a license to display the preprint in perpetuity. It is made available under a CC-BY 4.0 International license.

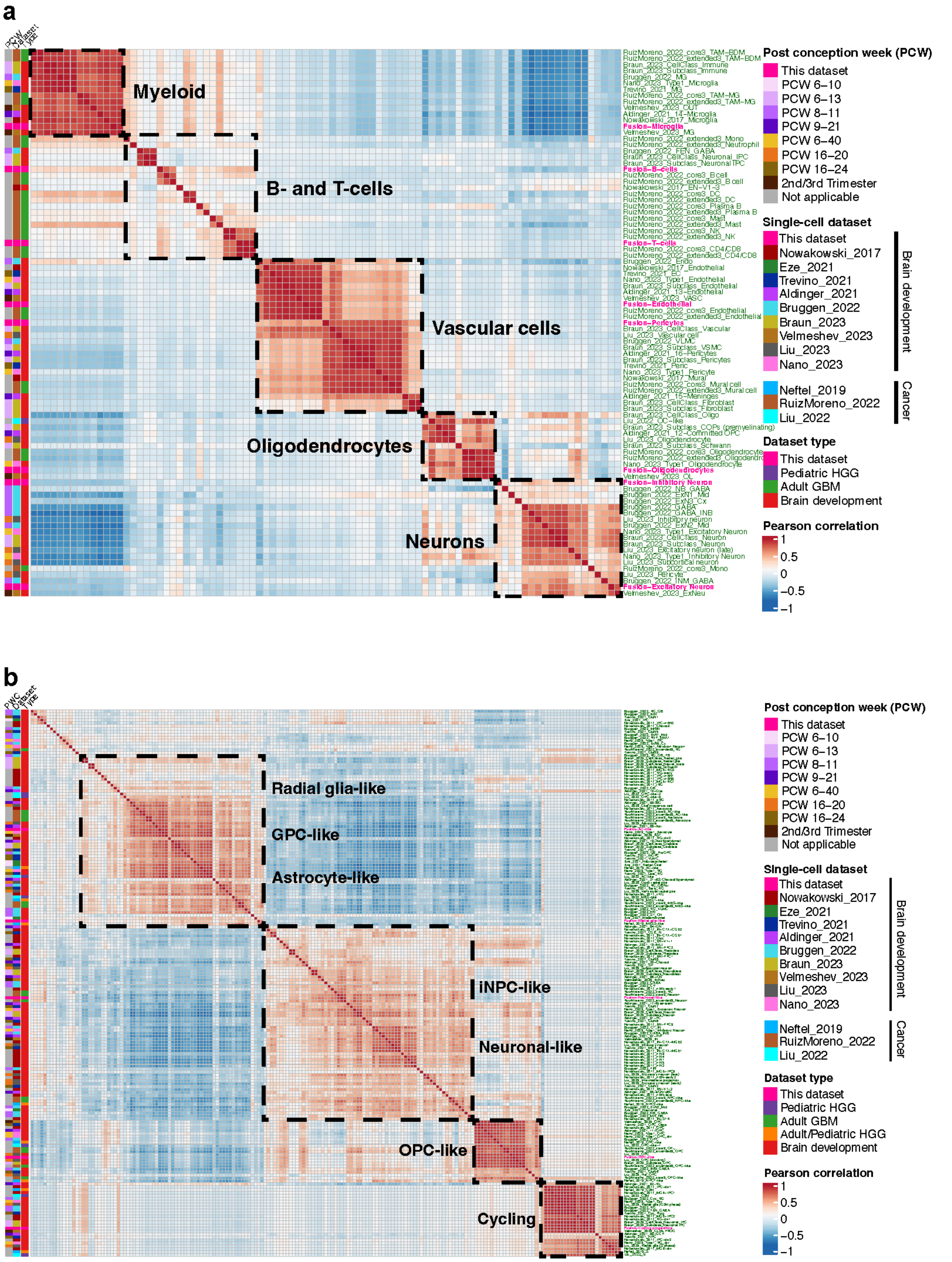

Figure S3– Single-cell data integration. Heatmap showing Pearson correlation scores between normal (a) and cancer (b) cell gene signatures from our dataset (pink labels) and published single-cell signatures of the developing brain and brain cancer (green labels).

bioRxiv preprint doi: https://doi.org/10.64898/2026.07.16.738862; this version posted July 16, 2026. The copyright holder for this preprint (which was not certified by peer review) is the author/funder, who has granted bioRxiv a license to display the preprint in perpetuity. It is made available under a CC-BY 4.0 International license.

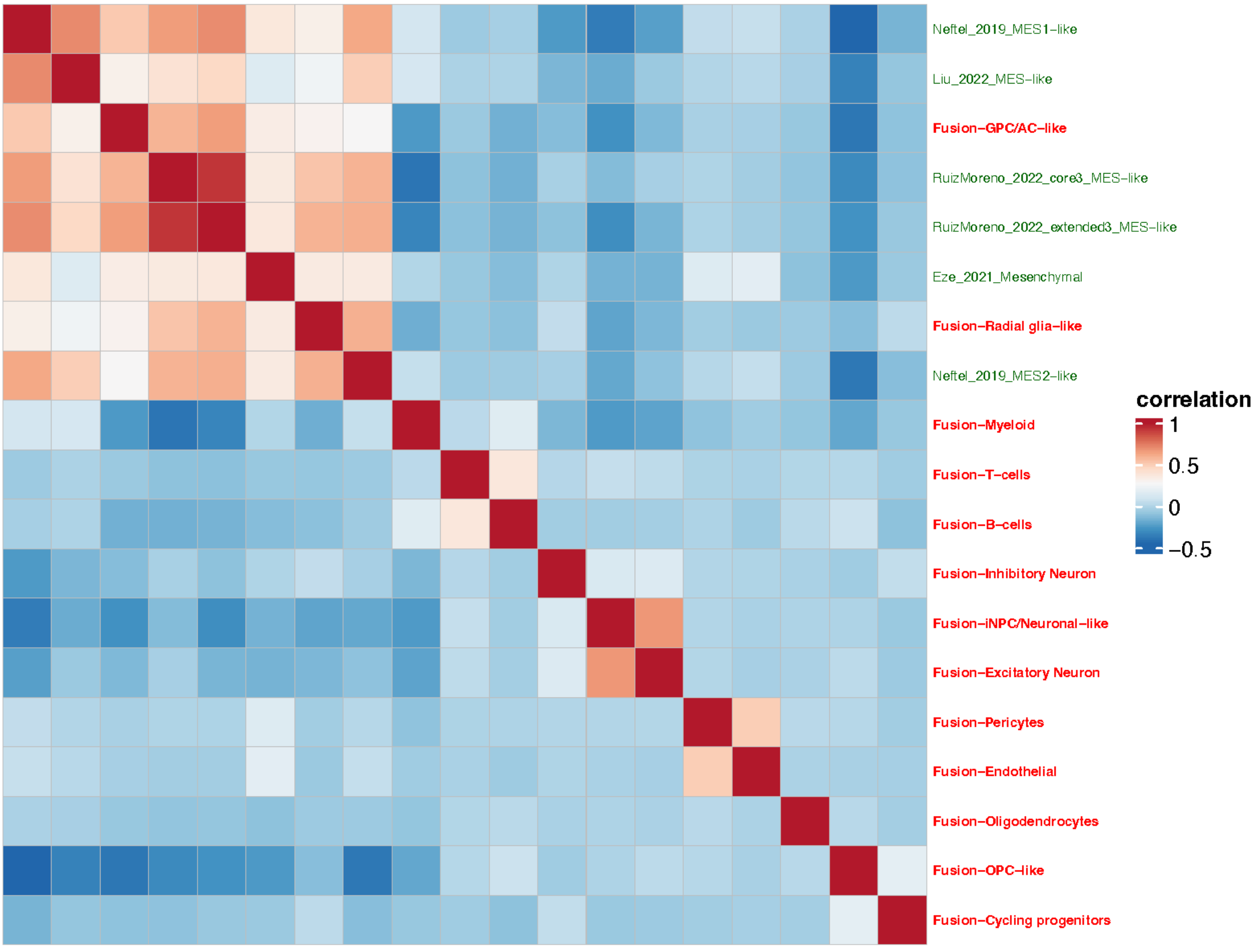

Figure S4– MES-like signature correlation. Heatmap showing Pearson correlation scores between all cells from our dataset (red labels) and MES-like populations from publicly available datasets (green labels).

bioRxiv preprint doi: https://doi.org/10.64898/2026.07.16.738862; this version posted July 16, 2026. The copyright holder for this preprint (which was not certified by peer review) is the author/funder, who has granted bioRxiv a license to display the preprint in perpetuity. It is made available under a CC-BY 4.0 International license.

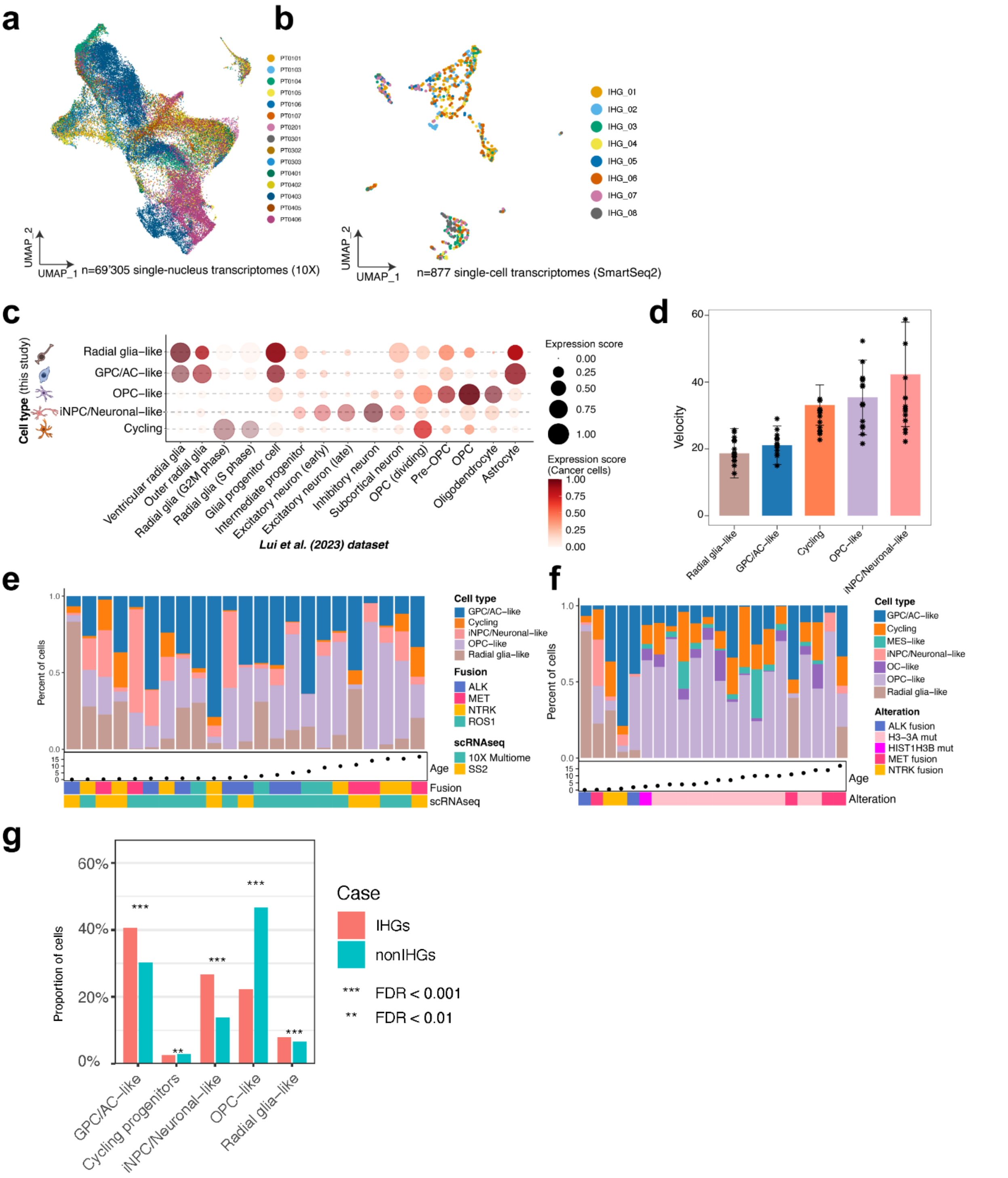

*Figure S5 – SmartSeq2 atlas of IHGs and cancer cell population heterogeneity. (a) UMAP of 69'305 single-nucleus transcriptomes colored by patient. (b) NMF-integrated UMAP of 877 cancer single-cells transcriptomes profiled by SmartSeq2 colored by patient. (c) Projection of cancer metaprograms onto Liu et al. (2023) developing brain single-cell atlas. Color scale: scores of normal cell signatures in cancer cells. Dot sizes: scores of cancer cell signatures in normal cells. (d) Average velocity value per sample and across cancer metaprograms. (e) Proportion of cancer cell types across the cohort (10X Multiome and SmartSeq2) and ordered by age. (f) Bar plot representing the proportion of tumor cell populations in SmartSeq2 datasets of RTK-driven gliomas (Fig. 2E) and H3K27M diffuse midline gliomas (Liu et al. (2024)). (g) Ratios of cell counts between the RG-like and GPC/AC-like populations and iNPC/Neuronal-like populations for n=8 infant patients ordered by age. Black bars for deceased patients. (h) Distinct cellular compositions of IHGs and non-IHG gliomas. Proportions of malignant cell populations identified by SmartSeq2 profiling in IHGs and non-IHG gliomas. Bars represent the percentage of cells belonging to each population within each tumor group. Statistical significance of differences in cell-state proportions between groups was assessed using differential proportion analysis with false discovery rate (FDR) correction; ***FDR < 0.001 and **FDR < 0.01.*

bioRxiv preprint doi: https://doi.org/10.64898/2026.07.16.738862; this version posted July 16, 2026. The copyright holder for this preprint (which was not certified by peer review) is the author/funder, who has granted bioRxiv a license to display the preprint in perpetuity. It is made available under a CC-BY 4.0 International license.

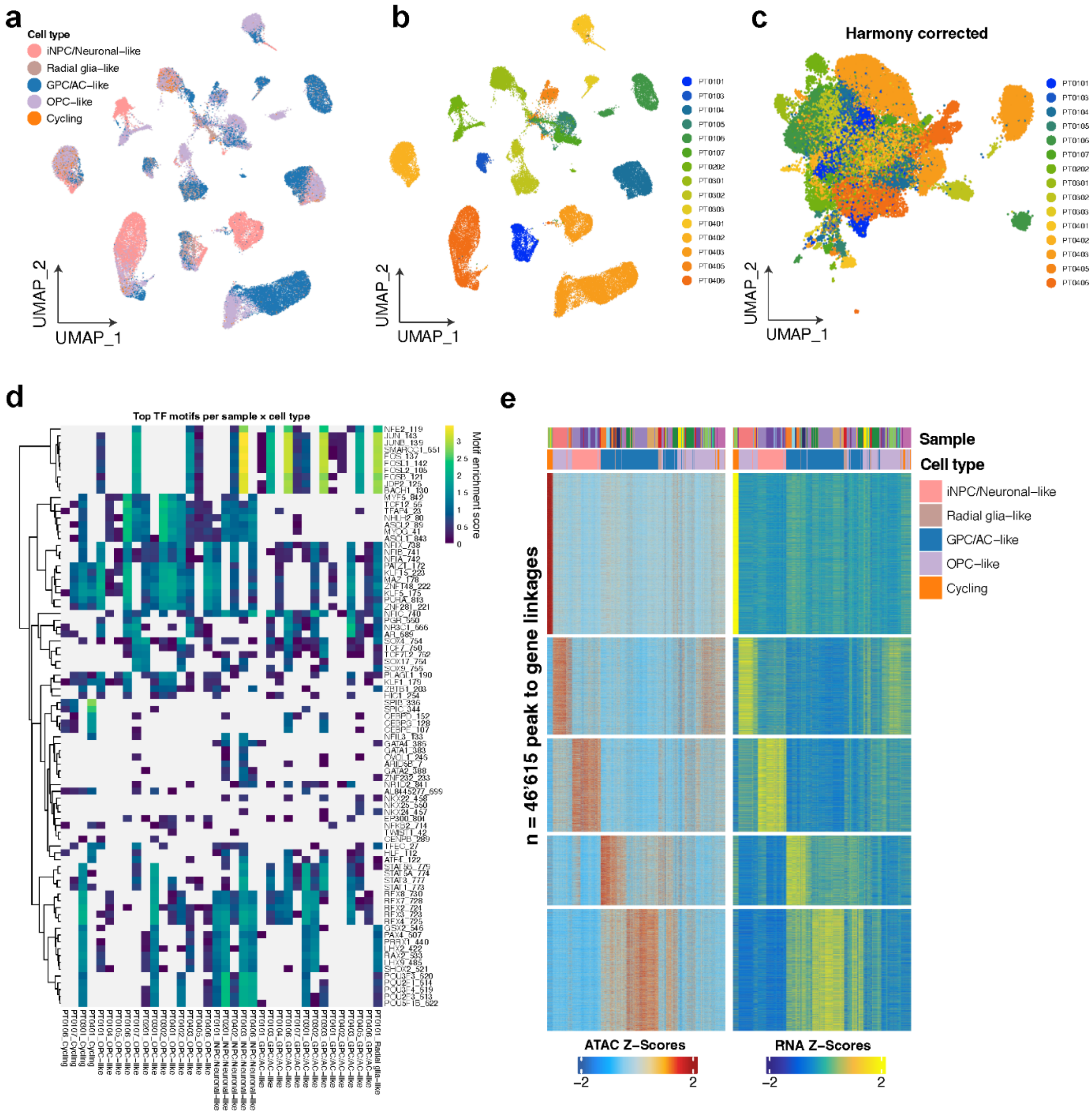

Figure S6– snATAC-seq atlas and chromatin accessibility analysis. (a) Unintegrated snATAC-seq atlas of n=15 patients colored by cancer cell type. (b) Unintegrated snATAC-seq atlas of n=15 patients colored by patient. (c) Harmony-corrected (integrated) snATAC-seq atlas of n=15 colored by patient. (d) Motif enrichment analysis on differential peaks across all patients and tumor cell type. Grey cells indicate motifs that were not detected. Only populations with > 30 cells were considered for this analysis. (e) Heatmap of peak-to-gene links displaying normalized gene expression and chromatin accessibility. A total of n=46'615 significantly linked peaks were identified. Paris (row) clustered using k-means (k=5). Legend represents the sample and annotated cell type.

bioRxiv preprint doi: https://doi.org/10.64898/2026.07.16.738862; this version posted July 16, 2026. The copyright holder for this preprint (which was not certified by peer review) is the author/funder, who has granted bioRxiv a license to display the preprint in perpetuity. It is made available under a CC-BY 4.0 International license.

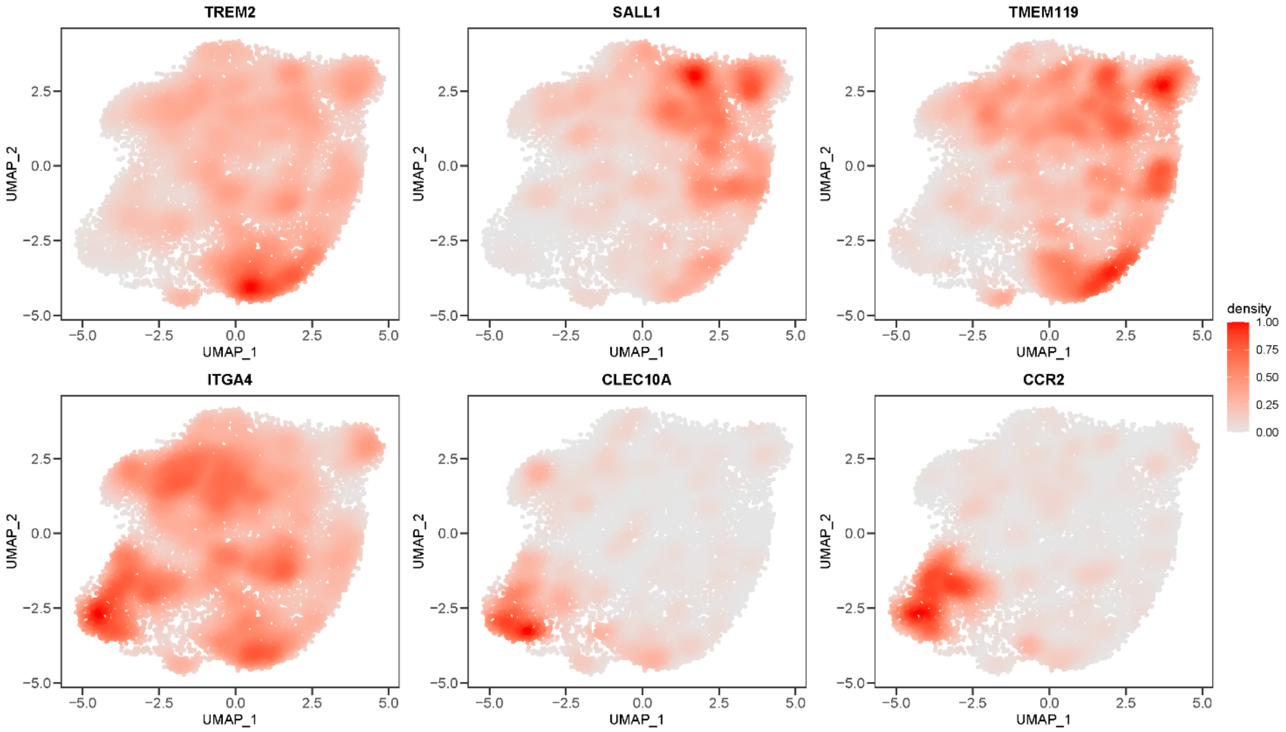

Figure S7– Tumor-associated macrophages. UMAP density plots of macroglia- and macrophage-associated transcripts.

bioRxiv preprint doi: https://doi.org/10.64898/2026.07.16.738862; this version posted July 16, 2026. The copyright holder for this preprint (which was not certified by peer review) is the author/funder, who has granted bioRxiv a license to display the preprint in perpetuity. It is made available under a CC-BY 4.0 International license.

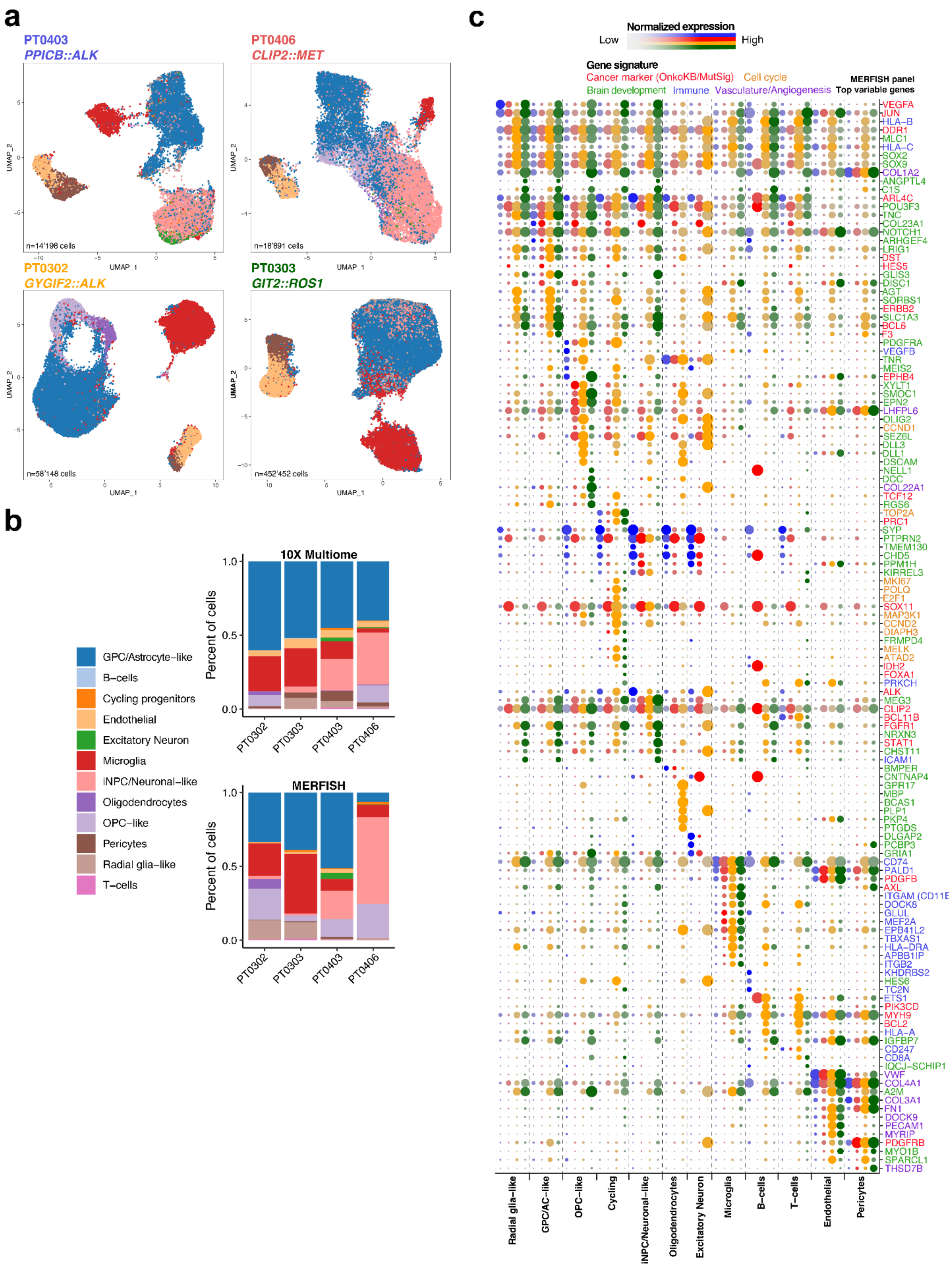

Figure S8– MERFISH patient atlases, cell type composition, and top variable genes. (a) UMAP of single cells resolved by MERFISH after QC filtering (n=500 genes) and colored cell type. (b) Proportions of cell types across patient samples, profiled by either 10X Multiome or MERFISH (inferred cell types for MERFISH only). (c) Dot plot of gene expression for top variable markers colored by patient (Fig. S6A) that were identified as differentially expressed across the tumor cell populations. Genes colored by marker type including cancer, brain development, cell cycle, immune, and vasculature, angiogenesis. Dot size indicate the percentage of cells expressing each per sample, while color intensity represents normalized expression level.
